# The Significance of Cancer Stem Cells and Epithelial–Mesenchymal Transition in Metastasis and Anti-Cancer Therapy

**DOI:** 10.3390/ijms24032555

**Published:** 2023-01-29

**Authors:** Lili Liang, Andreas M. Kaufmann

**Affiliations:** Department of Gynecology, Campus Virchow Klinikum, Charité-Universitätsmedizin Berlin, Corporate Member of Freie Universität Berlin, Humboldt-Universität zu Berlin, Berlin Institute of Health, 10117 Berlin, Germany

**Keywords:** tumor initiating cells, stemness, epithelial–mesenchymal transition (EMT), hybrid EMT, partial EMT, mesenchymal–epithelial transition (MET), tumor microenvironment (TME), invasion

## Abstract

Cancer stem cells (CSCs) have been identified and characterized in both hematopoietic and solid tumors. Their existence was first predicted by Virchow and Cohnheim in the 1870s. Later, many studies showed that CSCs can be identified and isolated by their expression of specific cell markers. The significance of CSCs with respect to tumor biology and anti-cancer treatment lies in their ability to maintain quiescence with very slow proliferation, indefinite self-renewal, differentiation, and trans-differentiation such as epithelial–mesenchymal transition (EMT) and its reverse process mesenchymal–epithelial transition (MET). The ability for detachment, migration, extra- and intravasation, invasion and thereby of completing all necessary steps of the metastatic cascade highlights their significance for metastasis. CSCs comprise the cancer cell populations responsible for tumor growth, resistance to therapies and cancer metastasis. In this review, the history of the CSC theory, their identification and characterization and their biology are described. The contribution of the CSC ability to undergo EMT for cancer metastasis is discussed. Recently, novel strategies for drug development have focused on the elimination of the CSCs specifically. The unique functional and molecular properties of CSCs are discussed as possible therapeutic vulnerabilities for the development of novel anti-metastasis treatments. Prospectively, this may provide precise personalized anti-cancer treatments with improved therapeutic efficiency with fewer side effects and leading to better prognosis.

## 1. Introduction

Metastasis is considered the primary cause of mortality in most cancer patients. It is associated with possible tumor recurrence. The involvement of CSCs in adjuvant therapy resistance and in the invasion–metastasis cascade has spurred interest in their biology and target ability by treatments. Conventional chemo/radiotherapies target rapidly proliferating cells and may result in initial tumor shrinking. Those treatments, however, may fail to prevent cancer recurrence due to the existence and characteristics of CSCs [1,2]. Despite great progress in the identification and understanding of CSC properties, many unsolved questions persist. Nonetheless, eradication of CSCs offers an exciting potential not only to eliminate the primary tumor, but also to obtain a long-term tumor-free period with the prevention of tumor metastasis. A key mechanism of metastasis is the epithelial–mesenchymal transition (EMT), its reverse process mesenchymal–epithelial transition (MET) and their hybrid or partial phase, allowing CSCs to migrate and seed into new distant sites [3].

In this review, we recapitulate the development of the CSC theory and discuss the CSC involvement in the metastatic cascade, with EMT/MET processes playing a crucial role. These findings lead to the assumption that the CSCs could be suitable therapeutic vulnerabilities for cancer treatments and predictors of metastasis and prognosis based on their functional and molecular properties.

## 2. Theory of CSCs and Their Characteristics

### 2.1. The Development of the CSC Theory and Their Discovery

**Figure 1:**
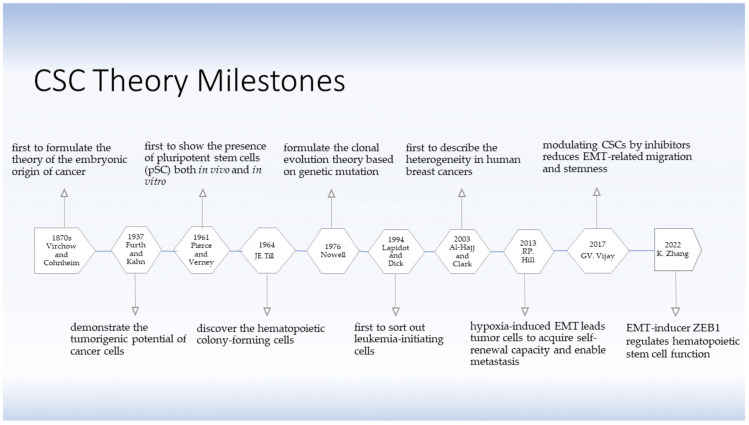
The development of the CSC theory. The theory of SCs and CSCs was initially conceived one and a half centuries ago. Virchow and Cohnheim first proposed the embryonic origin of cancer in 1870s. It took more than half a century until Furth and Kahn successfully established a mouse leukemia model indicating the possibility of tumor initiation by a minority of cancer cells. Since then, the CSC theory has been under challenge until more knowledge was accumulated. Today, the theory of CSCs has been substantiated and is regarded as reliable. CSCs have been demonstrated to be closely associated with EMT and metastasis [4,5,6,8,9,10,13,16,17,18].

The theory of the existence of stem cells (SCs) and CSCs was developed several decades ago. The CSC hypothesis applies the concept of SCs derived from embryogenesis to understand tumorigenic processes. As early as in the 1870s, Rudolph Virchow and Julius Cohnheim were the first to propose a theory on the embryonic-like origin of cancer, linking the similarities between neoplasms and embryonic tissue by what is known as CSCs now [4] (Figure 1). The theory did not attract much attention until 1937, when Furth and his colleagues intravenously inoculated small numbers of leukemic cells into mice in a limiting dilution experiment and found that a single malignant leukocyte was capable of producing leukemia, thereby describing the tumorigenic potential of certain tumor cells [5]. In 1961, Pierce and Verney discovered the multi-potentiality of ‘embryonal carcinoma cells’ from teratocarcinoma in both in vivo and in vitro experiments [6]. They demonstrated in 1964 that these ‘embryonal carcinoma cells’ were heterogeneous in their capabilities, giving strong support to the CSC theory [7]. In the same year, Till and his colleagues discovered that there were relative few hematopoietic ‘colony-forming cells’ present in hematopoietic tissue with capacity of extensive proliferation, differentiation and self-renewal [8]. Nowell stated the clonal evolution theory based on acquired genetic lability in 1976 [9], postulating that acquired genetic susceptibility allows sequential selection of variant sublines which are increasingly abnormal, explaining a stepwise tumor progression. In addition to hematologic malignancies [10], CSC-related investigations on solid tumors, including melanoma [11,12], breast cancer [13], colon cancer [14], and lung cancer [15], were performed. Benefitting from rapidly developing technologies, such as fluorescence-activated cell sorting (FACS) and magnetic-activated cell sorting (MACS), scientists continued to study more about CSCs. In 1994, Lapidot and Dick first sorted out leukemia-initiating cells based on the expression of cell surface markers, i.e., CD34^+^CD38^−^ cells, estimating that approximately one in a million leukemia cells were capable of tumor initiation [10]. In consistency, Al-Hajj and Clarke found that a minor proportion of breast cancer cells isolated as CD44^+^CD24^−/low^ lineage had the ability to form sustainable neoplasia, and based on this difference in cell surface markers, they distinguished the tumorigenic cancer cells from non-tumorigenic ones, describing for the first time the heterogeneity of human breast cancers in 2003 [13]. Taken together, these studies confirmed the existence of CSCs, consolidated the role and significance of CSCs in tumor initiation, development, growth, and recurrence, and supported the theory of CSCs concretely and reliably.

In 2013, Hill and his colleagues proposed that under hypoxia condition within the tumor microenvironment (TME), disseminated cancer cells acquired self-renewal capability during an EMT-enabled metastasis process. EMT induced loss of epithelial cell polarity and loss of epithelial markers, resulting in increased cell motility and promoting the maintenance of stemness [16]. In 2017, Vijay and her colleagues demonstrated that inhibition of glycogen synthase kinase β (GSK3β) decreased mesenchymal properties and downregulated the EMT-related migration. Further, inhibition of GSK3β significantly repressed the sphere formation, reduced stem cell properties and selectively killed mesenchymal cancer cells but not those with epithelial properties [17]. Zhu et al. demonstrated the EMT-inducer Zeb1 regulated the function of hematopoietic stem cells (HSCs) by suppressing the mitochondrial fusion [18]. Those supportive studies revealed the complex association of CSCs, EMT and metastasis.

### 2.2. The Characteristics of CSCs

Over the past decades, the existence of CSCs has been demonstrated in either hematological malignancies or solid tumors, including leukemia [10,19,20]^,^ breast cancer [13,21,22,23], bone cancer [24], brain cancers [25,26], colon cancer [14,27], head and neck squamous cell carcinoma (HNSCC) [28,29], lung cancer [15], liver cancer [30,31], melanoma [11], and ovarian cancer [32,33]. While most of the ground laying work on CSCs was performed in breast cancers, the principles are transferrable to other cancer types as well and are basic principles that can be applied in tumor biology of other solid tumors. In general, CSCs in different malignancies share certain characteristics.

#### 2.2.1. Self-Renewal and Aberrant Differentiation

Both normal SCs and CSCs possess self-renewal and differentiation capacities [34]. Generally, CSCs undergo asymmetric division with unequal cell size and distinct cell fates, i.e., one CSC generates a daughter CSC with unlimited proliferative potential through self-renewal, while it also generates a phenotypically distinct cancer cell with a limited lifespan and proliferative capacity which integrates into the tumor mass through differentiation-like mechanisms [35]. There have been different in vitro clonogenic assays, including sphere formation, organoid culture, and co-culture assays, rapidly developed to investigate the tumor heterogeneity and to address the potential of proliferation, differentiation, and self-renewal of CSCs. 

In earlier studies, a sphere-formation assay was established to study the neuronal stem cells from adult brain tissue and demonstrate the capacity of forming free-floating spheres in serum-free culture medium, called neurospheres [36]. Coherently, Wei et al. reported a population of human skeletal muscle cells that maintained their proliferative capacity and those cells expanded in vitro as myospheres for more than 20 passages, also termed myosphere-derived progenitor cells (MDPCs). The MDPCs expressed skeletal progenitor cell markers Pax7, ALDH1, Myod and Desmin, as well as stem cell markers Nanog, sex determining region Y-box2 (Sox2) and Oct3/4 [37]. Further, these cells were capable of spontaneous differentiation into myotubes and other mesodermal cell lineages, indicating the self-renewal and differentiation potential of SCs [37]. By investigating different tumor entities, we showed that those stem cell markers are shared by CSCs of different cancers [22,28,33]. By comparing a 2D-monolayer cell culture under normal adhesion and serum-containing culture conditions with a 3D-sphere culture under low-adherent and serum-free culture conditions, we observed that breast cancer stem cells (BCSCs) were enriched by growing out and forming spheroids in vitro with a higher enzyme activity of aldehyde dehydrogenase (ALDH) [22]. Additionally, in previous studies of HNSCC [28], oropharyngeal squamous cell carcinoma (OSCC) [38], and ovarian cancer [32,33], we demonstrated consistently that ALDH was a reliable marker for CSC identification. The ALDH^+^^/high^ cells were capable of indefinite self-renewal and tumor initiation by xenografting into animal models [39]. From our group, Xu et al. investigated the expression of the CSC marker ALDH1A1 in clinical specimen of HNSCC and OSCC. She found that ALDH1A1 was associated with poorly differentiated tumor histology independent of the patients’ HPV and smoking status, and patients with a higher frequency of ALDH1A1-expressing cells turned out to have a significantly shorter overall survival (OS) than those with a lower frequency. She also characterized the co-expression of ALDH1A1 and Twist1 in primary tumor and metastatic lymph node; however, no correlation was observed [40].

In addition, many cell markers, such as the epithelial cell-adhesion molecular (EpCAM), ESA, Nestin, disialoganglioside2 (GD2), CD47, CD49f, CD61, CD117, CD133, CD166, chemokine-activated G protein-coupled receptor4 (CXCR4) and its ligands CXCL12, and ATP-binding cassette-transporters G2 (ABCG2), are commonly expressed and shared as CSC markers by different solid tumor types [41]. Moreover, the well-established stemness conferring transcription factors (TFs) Nanog, Oct3/4, and Sox2 are applied in the CSC marker catalog, as they are involved in regulating stemness properties [34,41,42]. To further characterize the CSCs, additional protein markers associated with stemness and EMT have been reported, such as the overexpression of Sox9 and Snail in BCSCs [43,44,45]. 

#### 2.2.2. Quiescence/Dormancy

Targeting highly proliferative cells at different cell cycle phases, traditional chemo/radiotherapies induce DNA damage and trigger cell apoptosis. While CSCs can proliferate at a relatively rapid rate to repopulate the progeny pool in a fast-growing tumor, they can also switch into a quiescent state [46]. They have the ability to stay dormant and undivided for long periods until there are favorable conditions to re-stimulate their proliferation; therefore, during EMT and metastasis, CSCs display slow cycling features during dissemination [46,47] and can re-enter rapid proliferation in a new TME [35,48]. Quiescence, also termed dormancy occurs when cancer cells are alive, but their proliferation stops. It is a reversible status because quiescent CSCs can retain the capacity to return to a proliferative state. This indicates that within an invasion–metastasis cascade, CSCs will sustain in a quiescent phase during migration, trafficking through blood and/or lymph vessels and invasion into target site(s) and when adapting to a new TME in a distant organ [49]. It can explain the delayed recurrence of a tumor at the primary site or somewhere else in the body years after the first diagnosis [49,50]. 

**Figure 2:**
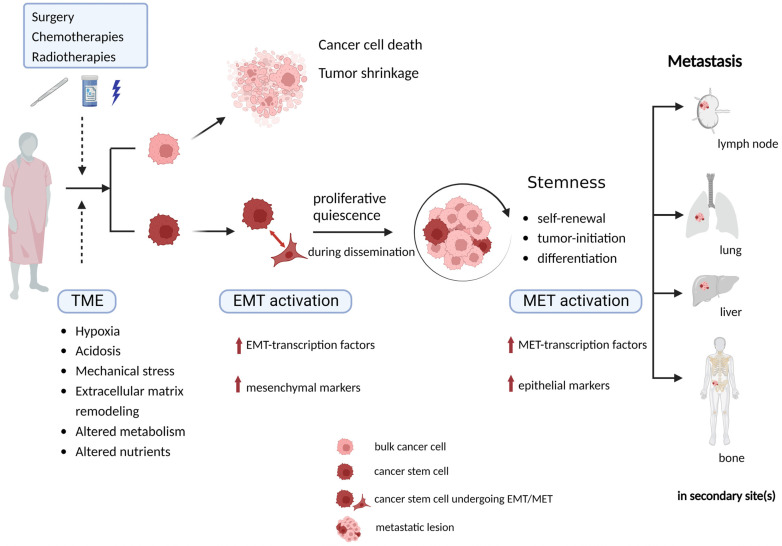
CSCs play a crucial role in metastasis due to their unique characteristics. Undergoing conventional standard anti-cancer therapies and the multi-directional influences from TME are cellular stress factors that CSCs are supposed to survive by activating an EMT program and stay in a quiescent state to keep themselves alive during dissemination and adjustment to different conditions in a new TME, being stimulated to proliferate when successfully adjusted to distant organs/sites. TME, tumor microenvironment; CSC, cancer stem cell; EMT, epithelial mesenchymal transition; MET, mesenchymal epithelial transition.

Quiescence is an important feature for CSC-related resistance to adjuvant therapies, under which CSCs remain arrested in G0/G1 phase of the cell cycle to survive and to undergo cellular reprogramming to adapt to the TME [1,46]. Moreover, the expression of some significant CSC markers and TFs, such as CD34, Nanog, Sox2, and Oct4 (also known as POU5F1), have only been reported to be elevated in some dormant cancer cells [46,51]. Benefiting from the flexible state-switching ability, CSCs are highly resistant to traditional chemotherapies and radiation therapies. Consequently, CSCs retain the proliferative capacity, metastatic potential and facilitate neoplastic regrowth and progression through repeated cycles of chemo-/radiotherapy [49]. In several solid tumors, such as colorectal cancer (CRC), pancreatic cancer, melanoma, and glioblastoma, a partial overlap has been identified between CSCs and the quiescent slow cycling cells [48]. As detailed above, CSCs can be resistant against standard anti-tumor therapies or under circumstances of, for example, hypoxia, by maintaining a quiescent state with minimal energy consumption and slow cell cycling [52]. In short, owing to their unique characteristics, CSCs play a crucial role in the metastatic context (Figure 2).

## 3. The Crosstalk between CSCs and EMT within the TME

### 3.1. TME and CSC

**Figure 3:**
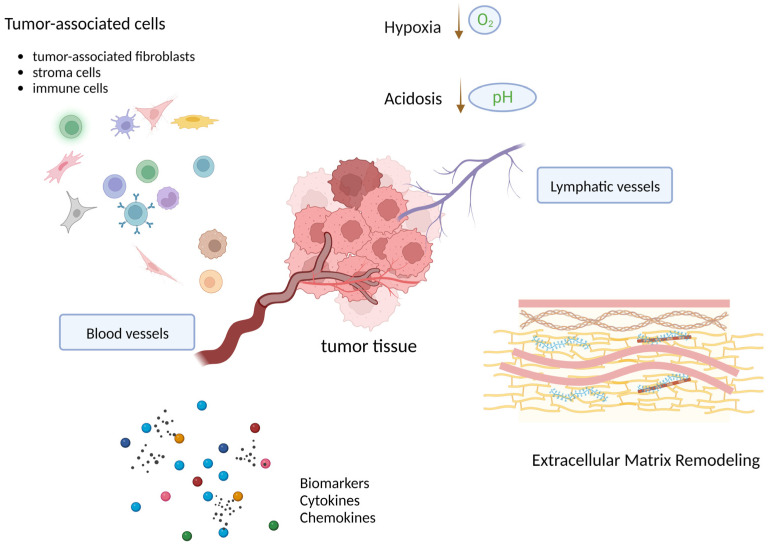
Interactions of CSCs within the TME. A tumor tissue is a complex composition of tumor cells and tumor-associated host tissue cells, along with blood and lymphatic vessels, molecules such as cytokines (colored circles), and acellular structures such as ECM. There are multi-directional interactions within the TME. These interactions alter that TME into a CSC supportive niche, which is tightly linked with the presence of hypoxia, acidosis, ECM remodeling, nutrients alterations and necrotic processes. The niche supports both the survival and maintenance of the CSCs by facilitating the promotion of stemness and regulation of dormancy.

Tumors are not isolated tissue masses of only proliferating cancer cells, but more as a complex community composed of tumor cells and tumor-associated cells, such as tumor-associated fibroblasts, stroma cells and immune cells, extracellular matrix (ECM)components and molecules, along with blood and lymphatic vessels, interacting with each other within the TME (Figure 3). There are exosomes and microvesicles, soluble factors such as growth factors, cytokines, and hormones included in the TME. All those biological, chemical, and mechanical factors provide CSCs with the necessary components to mediate the ECM remodeling and to regulate the proliferative and self-renewal signals by TFs and miRNAs to maintain stemness [53]. Cell-to-cell contacts are deconstructed through the decreased expression of E-cadherin, encoded by CDH1, leading to the loss of adherent junctions between cells, the conversion of cell morphology from a columnar shape to a spindle-like shape, and resulting in acquisition of motility [54,55]. 

Additionally, CSCs tend to reshape the TME into a supportive niche, which tightly links with the presence of hypoxia, acidosis, ECM remodeling, nutrient alterations, and necrotic processes. The niche functions to support both the survival and maintenance of the CSCs by facilitating the promotion of stemness and by regulating dormancy. Mariotto and her colleagues established an in vitro hypoxia-resistant breast cancer model and demonstrated that cycling hypoxic/re-oxygenation stress selected out a CD44^+^CD24^−^ESA^+^ subpopulation with sphere-forming capacity, which was sufficiently identified as BCSCs. In their study, the BCSCs were shown to survive stably under merely 1% O₂ condition by entering G0/G1 phase in a low metabolic state, the so-called dormancy [50]. Further, tumor-derived acidosis in the TME provides a strong evolutionary selection pressure and stimulates the emergence of aggressively invasive cancer cells [56]. Accumulated acidic metabolic products and accordingly cancer cell adaption to acidosis contributes together to ECM remodeling, local invasion, and ultimately tumor metastasis [56]. The most convincing clinical evidence is the effect of anti-acidosis drugs such as Proton Pump Inhibitors (PPIs). It has been shown that PPIs helped to improve the prognosis of patients receiving chemo/radiotherapy [57,58]. 

### 3.2. CSCs and EMT

EMT is a cellular program regulating trans-differentiation processes necessary for acquiring migratory capacity. It is crucial for embryogenesis such as gastrulation, neural crest formation, or heart development. It contributes not only to wound healing, but also to malignant progression and metastasis [59,60,61]. The EMT program has been presumed as the key mechanism by which cancer cells acquire stemness and the invasive properties necessary for migration, distant invasion, and metastatic outgrowth. Induction of the EMT program is mediated by different TFs and conditions in the TME.

#### 3.2.1. EMT/MET and Their Associated TFs 

**Figure 4:**
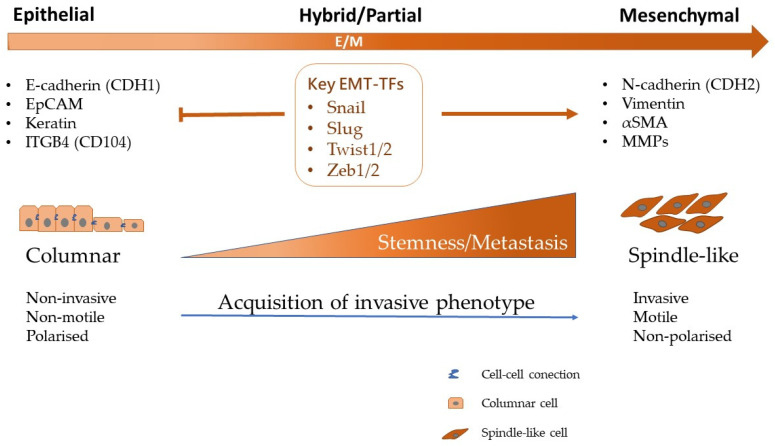
The EMT and its associated transcription factors. The EMT program requires the activation of a variety of TFs, including Snail and Slug, Zeb1 and Zeb2, Twist1 and Twist2, FOXC2 and GSC. It leads to the decreased expression of epithelial markers such as E-cadherin and increase the expression of mesenchymal markers such as N-cadherin. Accordingly, the morphologic and functional changes occur correspondently during the EMT process. The EMT regulation mediated by TFs has been proposed as a central key factor in the acquisition of stemness and promotion of metastasis. E, epithelial; M, mesenchymal; TFs, transcription factors; αSMA, alpha smooth muscle actin; MMPs, matrix metallopeptidases.

The EMT program provides genetic and cellular instructions for the first step of metastasis by generating migratory mesenchymal cells from immobile epithelial precursor cells [62]. As reported, EMT is associated with overexpression of specific TFs which regulate the structure of the cytoskeleton, cell polarity, cell-to-cell contact, and degradation/reorganization of the ECM [63]. Normally, epithelial cells are connected and attached to each other by tight junctions, adherens junctions, and desmosomes, while being anchored to the basement membrane by hemidesmosomes. During the classical EMT process, those TFs inhibit the expression of epithelial components while activating the genes involved in mesenchymal differentiation, leading to re-programming of the cell-to-cell interactions and cell-ECM interactions (Figure 4). EMT occurs at the beginning of the invasive process of epithelial tumors. These tumor cells undergoing EMT adopt features of mesenchymal cells while losing their epithelial traits, allowing cancer cells to migrate [54].

Shinya Yamanaka won his Nobel prize in 2012 after he successfully induced pluripotent stem cells (iPSCs) from mouse embryonic or adult fibroblasts by introducing four core TFs, Oct3/4, Sox2, c-Myc, and Klf4 [64]. In addition to these TFs, there are more TFs responsible for the EMT regulatory network in the TME, including FOXC2, and GSC, Snail (SNAI1) and Slug (SNAI2), Sox9, Twist1 and Twist2, Zinc finger E-box binding homeobox-1 (Zeb1) and Zeb2, together with a series of microRNAs (miRNAs) [65,66]. These TFs repress epithelial genes while directly or indirectly stimulating mesenchymal differentiation-associated genes [54]. Zhu et al. elucidated that Sox2 promoted the expression of Snail and stimulated the expression of mesenchymal markers N-cadherin and Vimentin in CRC cells but decreased the epithelial cell marker E-cadherin. The mesenchymal-like cancer cells also displayed greater proliferative activity, enhanced cell viability, increased motility, and sphere-formation capacity in vitro. They demonstrated that the activation of the Sox2-β-catenin/Beclin1/autophagy signaling axis stimulated the stemness, chemoresistance in CRC. Further, in in vivo xenograft models, inhibition of Sox2 restrained tumor growth and repressed chemoresistance. Together, those findings suggested that Sox2 plays a key role in promoting EMT process and stemness and contributes to chemoresistance in CRC cells [67]. Consistently, Chen and his colleagues demonstrated that the CRC cells with a high expression of Sox2 had stem-like features including a higher proportion of CD133^+^ expression, higher sphere formation efficacy, poorer differentiation, and greater migratory and invasive activity. They also reported that the patients with a higher Sox2 expression by the tumor cells had poorer outcome compared to patients with lower Sox2 expression [68]. 

The dynamic EMT-regulatory network determines the stem cell-like state. Guo and the colleagues identified that in order to maintain stem-like properties, both normal murine mammary stem cells (MaSCs) and human BCSCs share the same EMT program that is activated by Slug and Sox9 as major TFs [69]. Krebs et al. and Liu et al. demonstrated that Zeb1 expression strongly stimulates stemness, tumorigenicity, and tumor cell plasticity in pancreatic cancer; moreover, the epithelial-differentiated pancreatic cancer cells had a higher tumorigenic capacity when compared with mesenchymal-undifferentiated cells, in agreement with the view that EMT-MET dynamic transition reflects the stemness plasticity [70,71]. Induced by transforming growth factor-β (TGF-β), Zeb2 and Twist1 were both prominently upregulated during the EMT process in CRC cells. When Zeb2 and Twist1 were both inhibited, the metastasis was markedly suppressed in a mouse CRC model, suggesting that Zeb2 and Twist1 contribute to cancer cell migration [72]. Ye et al. demonstrated that the highest expression of Snail was found in the EpCAM¯ subpopulation, while increased Slug expression was detected in the EpCAM^+^ subpopulation. The mesenchymal markers, i.e., N-cadherin and Vimentin, were greatly increased while epithelial marker E-cadherin was decreased in the EpCAM^−^ subpopulation. They found that it was Snail but not Slug that was closely associated with the CSC phenotype [73].

The emergence of metastatic lesions in cancer patients can occur years after prior successful treatment of the primary tumor, but the mechanism is still poor understood. De Cock and her colleagues established a mouse model by cycling injection of highly metastatic murine mammary cancer cells. Thereby they induced solitary latent metastatic lesions localized in the mouse lung parenchyma and achieved a post-extravasation latent state in vivo. They induced the expression of Snail, Twist or Zeb1 in vivo and demonstrated that Zeb1 successfully stimulated latent disseminated tumor cells to enter a tumor-initiating state and then generated macro-metastasis. Those lung metastases were again collected, dissociated, and re-injected via the tail vein into mice. Subsequently, those mice also developed lung metastases, indicating that Zeb1 expression in vivo can activate EMT to facilitate the acquisition of tumor initiation and metastasis [74]. Meanwhile, those murine mammary cancer cells presented a phenotype CD29^+^CD24^−^, a stem cell-like state [74].

Within individual tumors, cancer cells display phenotypic heterogeneity in transition among epithelial (E), mesenchymal (M) and hybrid or partial-transitioned states [54,74]. Bierie et al. stratified the triple-negative breast cancer (TNBC) cells based on the expression of CD44, CD24 and the basal epithelial marker Integrin-β4 (ITGB4), an adhesion molecule. They found that ITGB4^+^ and ITGB4^−^ cells shared a common CD44^+^ phenotype. Interestingly, the ITGB4^+^ population existed within both CD24^+^and CD24^−^ cell populations, while the ITGB4^−^ population was primarily present in the CD24^−^ compartment. A minor subpopulation exhibiting an ITGB4^+^CD44^+^CD24^−^ phenotype, termed ITGB4^+^ CSCs, presented in a hybrid/partial E/M phenotypic state. These cells were prognostic markers for survival. In TNBC patients who received chemotherapy, the increased ITGB4 expression related to a worse 5-year relapse-free survival. Further they found that in the highly mesenchymal TNBC cell line SUM159, Zeb1 suppressed the expression of tumor protein63 isoform1 (TP63α), an epithelial TF that stimulates the expression of the ITGB4. Moreover, both the expression of Zeb1 and ITGB4 modulated towards more invasive histopathological phenotypes of tumors that derived from mesenchymal TNBC cells. Hence, the ITGB4 can be regarded as a prognostic marker to identify the more aggressive cancer subtype within TNBC [75].

#### 3.2.2. EMT Activation Regulates the Properties of CSCs

The activation of an EMT program allows CSCs to acquire certain mesenchymal features, while retaining certain epithelial cell determination [75]. Mani et al. demonstrated that the acquired EMT of BCSCs was associated with the acquisition of stem cell properties, including an increased ability for sphere formation and the expression of EMT markers. This illustrates a direct link between the CSCs and the EMT program [76]. CSCs can express EMT-associated markers, exhibiting features of E and/or M states, or a hybrid state; CSCs can re-acquire epithelial properties via MET, the reverse program, to initiate the growth of a secondary epithelial differentiated tumor [41]. Al-Hajj et al. were the first to demonstrate that a breast tumor consists of phenotypically distinct subpopulations of breast cancer cells. There was a minor subpopulation identified as the CD44^+^CD24^−^ cells, the BCSCs with the capacity to proliferate indefinitely to form new tumors. Such cells can be serially passaged indefinitely, indicating their self-renewal properties. The BCSCs were highly enriched in the ESA^+^CD44^+^CD24^−^ phenotype but not in ESA^−^CD44^+^CD24^−^ cells [13]. Consistently, Rodini et al. investigated oral squamous cell carcinoma cell lines and demonstrated that the ESA^high^CD44^high^ CSCs were more proliferative and enabled tumor growth, while the ESA^low^CD44^high^ CSCs were related to migration and invasion [77].

Studies have also shown that CSCs were important components of the circulating tumor cell (CTC) population, which play a crucial role in disseminating the tumor to remote site(s) [78]. Detection of CTCs offers potential to monitor tumor metastasis by liquid biopsy [79]. For example, CTCs can be positively selected with the help of antibodies to epithelial markers, i.e., anti-EpCAM and/or anti-cytokeratin, and/or antibodies to mesenchymal proteins, i.e., anti-Vimentin or anti-N-cadherin. Or CTCs can be selected negatively through depletion of leukocytes using anti-CD45 antibodies [80]. In addition, Markiewicz A et al. identified the molecular characteristics of CTCs with different epithelial, mesenchymal and CSC markers. They found that mesenchymal CTCs express significantly higher CD44, Vimentin, Nanog and Oct4, and induce more lymph node metastases and a larger tumor size. Importantly, the expression of Vimentin strongly correlated with invasion-related markers CXCR4 and uPAR, and stemness markers Nanog, Oct4 and ALDH1, indicating the close association with a higher risk of death in early breast cancer [78]. Their work consistently reported the close association between the mesenchymal CSC subpopulation within CTCs and metastasis. 

#### 3.2.3. Intra-/Extracellular Signals Regulating EMT and CSC

The acquisition of genetic alterations, the clonal evolution, and the TME contribute to the promotion of cancer invasion, metastasis, and therapy resistance. These events correspond to the heterogeneity of cancer cells. More evidence pointed out that the cells with stem-like phenotype in the individual cancers, the CSCs, is the main reason of resistance to therapies, tumor recurrence, and metastasis [61,81]. 

The transcription program switching among epithelial, mesenchymal and hybrid E/M states is induced by various signaling pathways that are mediated by TGF-β, bone morphogeneticprotein (BMP), Wnt-β-catenin, Notch, Hedgehog, receptor tyrosine kinases, and others [82,83]. These pathways are activated by various dynamic stimuli from the local microenvironment, including growth factors and cytokines, as well as hypoxia, and contact with the surrounding ECM [83]. Guen and his colleagues showed that sharing the same activation of the EMT program, basal MaSCs and BCSCs acquired their stemness properties by inducing primary epithelial ciliogenesis and Hedgehog (Hh) signaling activation. They elucidated that the mechanism connecting EMT programs and acquiring stemness was depended on primary cilia. The primary cilia were non-motile, cell-surface structures that served as platforms for receiving cues and enabled activation of various signaling pathways. Ablation of those primary cilia repressed the Hh signaling, the stemness of MaSCs, and the tumor-forming potential of BCSCs [84]. Coherently, increased expression of tetraspanin-8 (TSPAN8) in BCSCs promoted the expression of stemness markers Nanog, Oct4, and ALDH1, correlating with therapeutic resistance. Further, TSPAN8 inhibited the degradation of the Sonic-Hedgehog (SHH)/Patched1 complex through recruitment of the deubiquitinating enzyme ATXN3, resulting in the activation of Hh signaling and CSCs’ resistance to chemotherapeutic agents, and consequently enhanced tumor formation in mice. Accordingly, the expression levels of TSPAN8, SHH, Patched1, and ATXN3 were positively correlated in human breast cancer specimens, and the high expression levels of TSPAN8 and ATXN3 correlated with poor outcome of the patients [48]. Consistently, Rhim et al. found that the activity of the Hedgehog pathway was restricted to mesenchymal-derived stromal cancer cells. They also demonstrated that after the depletion of SHH, the pancreatic tumors were more aggressive with increased vascularity, enhanced proliferation features, and presenting histologic undifferentiated characteristics that were associated with poorer survival [85]. In short, various signals involved in the EMT program display multi-directional interactions and functions involving CSCs, consistent with the complexity of tumor cell regulation processes to maintain the growth, dissemination, and survival dynamically. The different signaling pathways controlling EMT through various regulatory networks, highlights opportunities to improve the prognosis for patients by targeting at CSCs.

## 4. CSC and EMT Involvement in the Metastatic Process

**Figure 5:**
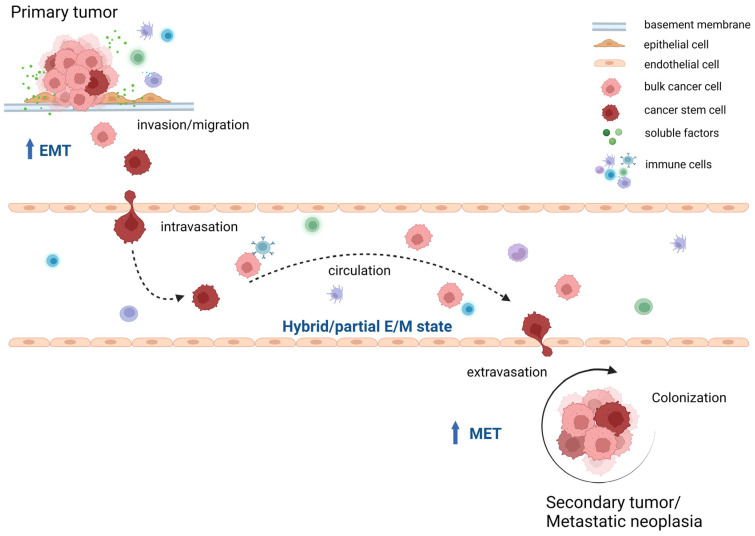
The invasion–metastasis cascade during cancer dissemination. Cancer cells acquire the necessary capabilities to complete the invasion–metastasis cascade. Firstly, the cancer cells at the primary tumor site spread into the surrounding tissue and pass through the epithelial basement membrane. Next, those cells successfully get access and invade into lymphatic and/or blood vessels (intravasation). These cells have to maintain cell viability during traveling in the circulation. When arriving at distant site(s), those disseminated cancer cells migrate out from the vessels (extravasation) to colonize in the target organ. Upon the process, small cell clones or disseminated cancer cells (micro-metastasis) must survive and finally, adjust to the new microenvironment, proliferate and form macroscopic metastasis (macro-metastasis). E, epithelial; M, mesenchymal; EMT, epithelial–mesenchymal transition; MET, mesenchymal–epithelial transition.

Metastasis is responsible for 90% of cancer-related mortality [39] and has been considered as a late stage in cancer progression when the cancer cells have acquired the necessary capabilities to complete the invasion–metastasis cascade [46]. To complete this cascade, the EMT program regulates the whole process. The EMT facilitates the dispersion of cancer cells from primary tumor site, the ECM remodeling, invasion into the surrounding tissue parenchyma towards lymphatic and/or blood vessels. Cancer cells undergo trans-endothelial migration to penetrate into a vessel (intravasation). Such cancer cells then have to stay alive in the circulation. When arriving at distant sites, the disseminated cancer cells have to penetrate again out from the vessels (extravasation), switching then their phenotype via a MET program to colonize successfully [83]. Upon the process, small cell clones or individual disseminated cancer cells (micro-metastasis) must maintain their survival and must adjust to proliferate in the very microenvironment of a distant organ/site to grow to macroscopic metastasis [86] (Figure 5). 

The metastasis continues even after the primary tumor is surgically removed and/or after standard chemo/radiotherapies. Studies have emphasized the role of CSC and the presence of circulating tumor cells (CTCs) [39,86,87]. It has been hypothesized that merely a small subpopulation of cancer cells is capable of completing the entire process of the invasion–metastasis cascade. Those cells designated as metastasis-initiating cells (MICs)/CTCs, overlap with the CSC population [88,89]. EMT has a crucial impact on the recovery of mesenchymal CTCs when settling down in a new TME [89]. Driven by EMT-TFs, such as Snail, Slug and Zeb1, the transition of CSCs from the non-motile epithelial phenotype to a more motile mesenchymal phenotype, i.e., potential mesenchymal MICs, promotes cancer dissemination, the early phase of metastasis [41]. Undergoing EMT process, the epithelial CSCs lose cell polarity with decreased expression of the adhesion associated molecules and increased invasive potentials, turn into mesenchymal metastatic CSCs. After efficiently spreading to a distant site, those cells undergo a converting transition from quiescent mesenchymal to an actively proliferating epithelial phenotype with a proliferation ability for rapid re-growth, described as phenotypic plasticity [44,90]. 

Distinct phenotypes of CSC subpopulations account for tumor growth and metastatic activity [39,91,92]. As demonstrated, most of the CD44^+^CD24^−^ BCSCs were located at the invasive edge of the tumor mass, indicating that their presence is associated with enhanced aggressive invasiveness [13]. Wicha and his colleagues found that BCSCs existed in distinct mesenchymal-like and epithelial-like states. The quiescent mesenchymal BCSCs, characterized as CD44^+^CD24^−^ phenotype, were located at the infiltrating edge of the tumor, whereas epithelial BCSCs, characterized as ALDH^+^ phenotype, proliferated more actively in a more central area [90]. Accordingly, in clinical specimen of breast cancer patients, the specific EMT markers, such as Vimentin, Zeb1, Twist1, β-catenin, and MMP9 showed opposite expression patterns between the CD44^+^CD24^−^ phenotype and the ALDH^+^ phenotype [90]. Tumor cells can convert their phenotype back and forth interchangeably among an epithelial, mesenchymal, and hybrid state [46]. In that intermediate status, tumor cells present certain mesenchymal as well as partial epithelial features, which makes them flexible in adapting to a new TME. 

## 5. Therapeutic Implications for Targeting at CSC

The existence of CSCs within a tumor provides an explanation for clinical treatment failures in patients with cancer. CSCs can be stimulated by the therapeutic pressure, as well as changes and/or stimuli within the TME, and display a high phenotypic plasticity, accordingly, with distinct functional appearances [93]. CSCs are closely involved in tumor initiation, development, metastasis, and recurrence due to their intrinsic stemness properties and their ability to undergo the EMT process. It is reasonable to regard CSCs as a potential therapeutic target to prevent metastasis and to revert the chemo-/radioresistence. Yang et al. showed that ALDH^+^ BCSCs were responsible for cisplatin resistance and the ALDH^+^ BCSCs displayed higher levels of reactive oxygen species (ROS) from bulk breast cancer cells. He demonstrated that the anti-alcoholism drug disulfiram (DSF) inhibited the activity of the enzyme ALDH as well as stem cell-like properties in the ALDH^+^ BCSCs. He further demonstrated that DSF treatment modulated ROS accumulation and helped to overcome cisplatin-resistance synergistically by enhancing the cytotoxic effect of cisplatin on the ALDH^+^ BCSCs and their cisplatin-resistant variant sublines, indicating that DSF can sensitize CSCs to chemotherapy [22]. Phillips et al. demonstrated that the breast cancer MCF7 cells with CD44^+^CD24^−^ phenotype were relatively resistant to radiotherapy, generating less ROS and had decreased DNA damage in response to radiation, leading to increased cancer cell number after short courses of fractionated irradiation [94]. 

CSCs change their characteristics on both the genetic and protein level in respond to the various signaling networks in the TME by activating the EMT/MET process [83]. Considering the crucial role of CSCs involved in the EMT and metastatic process, a new window may open to study the biology of metastasis with emphasis on their importance for understanding and preventing metastasis. Strategies for CSC-targeted therapies include targeting CSC markers and EMT-related markers, and modulating of intra-/extra-cellular signals that regulate EMT and CSCs, such as TGF-β, Wnt, and β-catenin [93]. Some pre-clinical experiments targeting EpCAM^+^ CSCs have been performed, i.e., Zhou et al. assessed EpCAM-targeting CAR-T cells and their killing efficiency in vitro by co-culture with colon cancer cell lines and demonstrated that EpCAM-CAR-T cells exhibited a significantly higher apoptotic effect on cancer cells compared with non-EpCAM-modified T cells [95]. Zhang et al. showed that EpCAM-CAR-T-cell treatment significantly restrained colorectal tumor growth and formation in mouse xenograft models [96]. In addition, the therapies targeting at CD133^+^ CSCs have shown promising results either as monotherapy or by using multiple therapeutic approaches in combination with cytostatic agents [93]. In a phase I clinical trial, the 23 patients with hepatocellular carcinoma received CD133-directed CAR-T cell infusions, and 21 patients had not developed detectable de novo lesions within the observation time frame; thereby, the trial demonstrated feasibility, safety, and efficacy of CD133-directed CAR T-cell therapy in patients [93]. Those pre-clinical and clinical trials provide a future perspective for the cancer treatments targeting CSCs.

## 6. Conclusions

CSCs are closely involved in tumor initiation, development, metastasis, and recurrence due to their intrinsic stemness properties and capacity to undergo the EMT/MET transition. Therapies fail to achieve CSC clearance due to their reversible phenotypic plasticity. The quiescent mesenchymal CSCs stay alive during dissemination, and they can retain the capacity to re-enter a proliferative epithelial state when adapting to a new TME. Understanding the significance of CSCs undergoing EMT for the invasion–metastasis cascade and demonstrating how CSCs facilitate tumor metastasis remain important and necessary to be elucidated. Potentially, eradicating CSCs or blocking the CSC-associated EMT process will prevent metastasis. Precise personalized anti-cancer treatments can be imagined to improve therapeutic efficiency with fewer side effects, leading to less metastasis and better prognosis.
